# XGBPRH: Prediction of Binding Hot Spots at Protein–RNA Interfaces Utilizing Extreme Gradient Boosting

**DOI:** 10.3390/genes10030242

**Published:** 2019-03-21

**Authors:** Lei Deng, Yuanchao Sui, Jingpu Zhang

**Affiliations:** 1 School of Computer Science and Engineering, Central South University, Changsha 410075, China; leideng@csu.edu.cn (L.D.); suiyuanchao@csu.edu.cn (Y.S.); 2 School of Computer and Data Science, Henan University of Urban Construction, Pingdingshan 467000, China

**Keywords:** hot spots, protein–RNA interfaces, XGBoost, two-step feature selection

## Abstract

Hot spot residues at protein–RNA complexes are vitally important for investigating the underlying molecular recognition mechanism. Accurately identifying protein–RNA binding hot spots is critical for drug designing and protein engineering. Although some progress has been made by utilizing various available features and a series of machine learning approaches, these methods are still in the infant stage. In this paper, we present a new computational method named XGBPRH, which is based on an eXtreme Gradient Boosting (XGBoost) algorithm and can effectively predict hot spot residues in protein–RNA interfaces utilizing an optimal set of properties. Firstly, we download 47 protein–RNA complexes and calculate a total of 156 sequence, structure, exposure, and network features. Next, we adopt a two-step feature selection algorithm to extract a combination of 6 optimal features from the combination of these 156 features. Compared with the state-of-the-art approaches, XGBPRH achieves better performances with an area under the ROC curve (AUC) score of 0.817 and an F1-score of 0.802 on the independent test set. Meanwhile, we also apply XGBPRH to two case studies. The results demonstrate that the method can effectively identify novel energy hotspots.

## 1. Introduction

The proteins and nucleic acids constitute the two most important types of biological diversity in living organisms, and they each have their structural characteristics and particular fixed function. Protein–RNA interaction site prediction is of great significance and helps us understand how protein function is achieved so that we can better understand and study the various features of cells [1,2,3,4,5]. Among the protein–RNA interface residues, as is known to all, only a small number of hot spots are essential for the binding free energy. Sufficient identification of these hot spots helps to better understand the molecular mechanisms. Moreover, the interactions of protein with small molecule compounds are the basis for drug design, and structure-based drug design has achieved great success in the development of drugs [6,7]. In recent years, the success rate of the discovery of lead compounds by the molecular docking of compound databases and protein structures has significantly improved. The precise localization of hot spots can elucidate the principle of protein–RNA interactions and provide a very significant theoretical support and a basis for target drug preparation. At present, the research of protein–RNA binding and the critical hot spots in protein–RNA interfaces is an important research direction of bioinformatics and cell biology [8,9].

The characteristics for determining protein–protein binding hot spots have been extensively studied. The research proves that the composition at amino acid in hot spot areas differ from that in non-hot spot areas. For example, Thorn and Bogan [10] found that hot spots are abundant in Arg, Tyr, and Trp because of their conformation and size. Meanwhile, they proved that hot spots are concerned with energetically less essential interfaces, whose O-ring shape seems to occlude bulk water molecules from the hot spots. Furthermore, analysis has demonstrated that Asp and Asn are more common in hot spots than Glu and Gln because of the differences in side-chain conformational entropy. In recent years, a variety of machine learning algorithms have been used to predict protein–protein interaction hot spots with structural and sequence properties [11,12,13,14,15,16]. However, these protein–protein interaction hot spot prediction methods and features cannot be directly used to predict protein–RNA binding hot spots. So far, only a few methods have been used to predict protein–RNA interaction hotspots. Barik et al. proposed HotSPRing [17] to identify the hot spots with physico-chemical and structural features in protein–RNA complexes using random forest classifiers. Pan et al. proposed a new method named PrabHot (Prediction of protein–RNA binding hot spots) [18], which used an ensemble of conceptually distinct machine learning algorithms to predict the hot spots.

**Figure 1:**
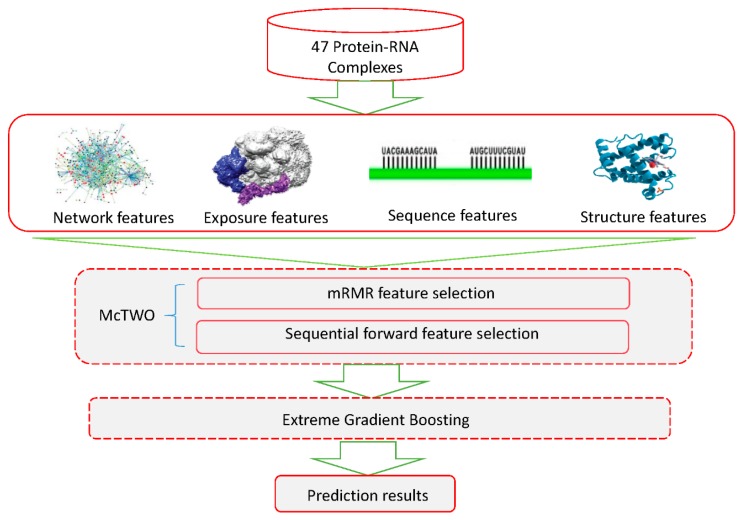
Flowchart of XGBPRH method. The experimental dataset of 47 protein–RNA complexes comes from Pan et al.’s work [18]. We extracted 156 network, exposure, sequence, and structure features. We then adopted the McTWO feature selection algorithm to select the optimal features and used the selected optimal features to train the eXtreme Gradient Boosting (XGBoost) classifier. Finally, we evaluated the performance on the training dataset and independent dataset.

In this paper, we propose XGBPRH, a powerful computational method to identify hot spots in protein–RNA complexes. First, 156 exposure (solvent exposure), network (residue interaction network) [19], structure [20,21], and sequence features are extracted. To remove irrelevant and redundant information, we use an McTWO feature selection algorithm on the 156 features to select six optimal features. Then, the six optimal features are fed into an eXtreme Gradient Boosting (XGBoost) classifier [22] for predicting protein–RNA binding hot spots. We also evaluate the relative importance of the six optimal features. The results show that exposure and network features are crucial for prediction. Furthermore, we compare XGBPRH with two recent methods, namely HotSPRing and PrabHot, using an independent dataset. The experiments demonstrate that XGBPRH gains the highest values of *F_1_* and area under the ROC curve (AUC), respectively, which are significantly higher than those of the other two methods. The flowchart of XGBPRH is depicted in the following Figure 1.

## 2. Materials and Methods

### 2.1. Datasets

In this study, the experimental dataset was derived from the works of Barik et al. [17] and Pan et al. [18]. It includes 63 protein–RNA complexes. After removing the redundancy [23] with sequence similarity greater than 40% by using CD-HIT, a dataset of 47 protein–RNA complexes was obtained. Usually, protein–RNA complexes whose corresponding binding free energy change (∆∆ G) ≥1.0 kcal/mol are termed as hot spots, and the remaining residues are considered as non-hot spots. Based on this definition, 102 energetically unimportant residues (negative samples) and 107 hot spots (positive samples) were curated from the 47 complexes. Meanwhile, the structural and sequence information of RNAs and proteins in complexes were obtained from the Protein Data Bank (PDB) [24]. The 47 complexes were randomly split into a training benchmark dataset and an independent testing dataset (Table 1). The training dataset has 32 protein–RNA complexes and the independent dataset has 15 complexes. The source code used on this analysis and datasets used are available online at https://github.com/SupermanVip/XGBPRH.

### 2.2. Performance Evaluation

In order to evaluate the performance, we chose the following seven evaluation metrics, which mainly include specificity (SPEC), sensitivity (recall/SENS), F1-score (F1), precision (PRE), the area under the ROC curve (AUC), accuracy (ACC), and the Matthew’s correlation coefficient (MCC). These metrics are termed as follows:

SPEC = TN/(TN + FP)(1)

SENS = TP/(TP + FN)(2)

F1 = 2 × Recall × Precision/ (Recall + Precision) (3)

PRE = TP/(TP + FP)(4)

AUC = P(P_positive_ > P_negative_) (5)

ACC = (TP + TN)/(TP + TN + FP + FN)(6)

(7) $$\mathrm{MCC}=(\mathrm{TP}\times \mathrm{TN}-\mathrm{FP}\times \mathrm{FN})/\sqrt{\left(\mathrm{TP}+\mathrm{FP}\right)\left(\mathrm{TP}+\mathrm{FN}\right)\left(\mathrm{TN}+\mathrm{FP}\right)\left(\mathrm{TN}+\mathrm{FN}\right)}.$$

### 2.3. Feature Extraction

Extracting effective features is the key to improving classification performance [25,26,27,28,29]. We initially calculated a combination of 156 features, including exposure features, network features, structural and sequence features. Thirty-one of 156 features were newly curated, and the remaining features were extracted from Pan’s work [18]. Details of these features are as follows.

#### 2.3.1. Features Based on Network

According to spatial distance or interaction energy, a residue interaction network (RIN) that captures the inter-residue interactions was obtained. A combination of seven topological features of the RIN were calculated using the NAPS tool [30]: degree, closeness, eigenvector centrality, betweenness, clustering coefficient, average nearest neighbor degree, and eccentricity.

Degree represents the number of direct neighbors of a node, which is defined as

(8) $$C_{d}\left(u\right)=\underset{v\in V}{\sum }A_{uv}$$

where *A_uv_* is the number of contacts between nodes *v* and *u*, and *V* is the set of all nodes.

Closeness is a centrality measure of a node and is termed as the inverse of the shortest path distance of the node to all other nodes in the network.

(9) $$C_{cl}\left(u\right)=\left(n-1\right)/\underset{v\in V}{\sum }dist_{uv}$$

Here, *dist_uv_* is the shortest path distance between nodes *u* and *v*.

Betweenness is termed as the ratio of all the shortest paths passing through a node and the total number of shortest paths in the network.

(10) $$C_{b}\left(u\right)=\underset{s\neq u\in V}{\sum }\underset{t\neq u\in V}{\sum }\sigma_{st}\left(u\right)/\sigma_{st}$$

where *σ_st_*(*u*) is the total number of shortest paths between nodes *s* and *t* passing through node *u*, and *σ_st_* is the number of shortest paths between nodes *s* and *t*.

The clustering coefficient is defined as the ratio of numbers of connected neighbors of a node to the total number of connections possible between the neighbors. It is a measure of the closeness of the neighbors of a node.

(11) $$C_{cc}\left(u\right)=\lambda \left(u\right)/\gamma \left(u\right)$$

where *λ*(*u*) is the neighbors of *u* connected by an edge, and γ(u) is defined as follows:

(12) $$\gamma \left(u\right)=C_{d}\left(u\right)\left(C_{d}\left(u\right)-1\right)/2.$$

The eccentricity indicates the distance from the shortest path of the node to the farthest node in the network:

(13) $$C_{e}\left(u\right)=max\left(dist\left(u,v\right)\right).$$

#### 2.3.2. Features Based on Solvent Exposure

The solvent exposure measures to what extent a residue is accessible to the solvent (usually water) surrounding the protein. It is crucial for understanding the structure and function of the protein. We used a new 2D exposure measure, a half-sphere exposure (HSE) [31], which divides a residue’s sphere into two half spheres: HSE-down and HSE-up. We employed HSEpred [32] to calculate the structure information, including HSE-down, CN (coordination number), and HSE-up. Moreover, the exposure features including HSEAD (number of *C_a_* atoms in the lower sphere), HSEAU (number of *C_a_* atoms in the upper sphere), HSEBD (the number of *C_β_* atoms in the lower half sphere), HSEBU (the number of *C_β_* atoms in the upper sphere), RDa (*C_a_* atom depth), and RD (residue depth) were calculated using the hsexpo program [31].

#### 2.3.3. Features Based on 3D Structure

A protein 3D structure refers to a polypeptide chain that is further coiled and folded on the basis of various secondary structures to form a specific spatial structure. Structure-based features have been widely use to predict protein interaction sites [33].

The solubility and stability of proteins are affected by the surface interacting macromolecules in the form of solvents and small solutes in solution. Consequently, the macromolecular surface is an important factor for researching the structure and function of molecules. We considered the surface curvature and the molecular surface area, and employed Surface Racer [34] to calculate these two characteristics. We also calculated the total solvent accessible surface area, the framework solvent accessible surface area, the total associated solvent accessible surface area, the backbone relative solvent accessible surface area, the average depth index, the maximum depth index, the average protrusion index, the maximum protrusion index, and the hydrophobicity through PSAIA [35].

#### 2.3.4. Features Based on Protein Structure

The protein structure features were also commonly used, and they are as follows:

1. Solvent accessible area (ASA). ASA represents the relatively accessible surface area, which can be calculated using the Naccess [36] program. These ASA features include values of all atoms (ASA_aaa), relative all atoms (ASA_raa), absolute total side (ASA_ats), and relative total side (ASA_rts). We also computed the ΔASA (the change in the solvent accessible surface area of the protein structure between bound and unbound states).
2. Secondary structure. We calculated seven secondary structure features: the residue number of first bridge partner, the solvent accessible surface area, *C_α_* atom dihedral, peptide backbone torsion angles, and bend angles through DSSP [37] and SPIDER2 [38].
3. Four-body statistical pseudo-potential (FBS2P). The FBS2P score, which is based on the Delaunay tessellation of proteins [39], can be written as the following formula.
  (14) $$O_{ijpq}^{\alpha }=\log\left(\frac{f_{ijpq}^{\alpha }}{P_{ijpq}^{\alpha }}\right)$$
  where *i*, *j*, *p*, and *q* are termed as the four amino acids in a Delaunay tetrahedron of the protein. $f_{ijpq}^{\alpha }$ represents the observed frequency of the residue component (*ijpq*) in a tetrahedron of type *a* over a set of protein structures, and $P_{ijpq}^{\alpha }$ represents the expected random frequency.
4. Energy scores. We used ENDES [40] to calculate seven energy scores: residue energy (Enrich_re), side-chain energy (Enrich_se), conservation (Enrich_conserv), two combined scores (Enrich_com1 and Enrich_com2), relative solvent accessibility (Enrich_rsa), and interface propensity (Enrich_ip).
5. Hydrogen bonds. The hydrogen bonds were calculated using HBPLUS [41].
6. Helix and sheet. The features of α-helix and β-sheet secondary structure are represented with one-hot encoding [42].

#### 2.3.5. Features Based on Protein Sequence

Besides some common features, we selected a few novel features such as backbone flexibility and side-chain environment. These features can be detailed as follows:

1. Backbone flexibility. The protein is flexible and has a range of motion, especially when looking at intrinsically disordered proteins. The feature is calculated by DynaMine [43].
2. Side-chain environment. The side-chain environment (pKa) represents an effective metric in determining the environmental characteristics of a protein. The value of pKa was acquired from Nelson and Cox, indicating a protein side-chain environmental factor, and has been utilized in previous research.
3. Position-specific scoring matrices (PSSMs). The scoring matrices can be calculated by PSI-BLAST [44].
4. Local structural entropy (LSE). LSE [45] is described as the degree of conformational heterogeneity in short protein sequences.
5. Conservation score. We mainly used Jensen–Shannon divergence [46] to calculate the conservation score, which is calculated as follows:
  (15) $${\mathrm{Score}}_{i}=-\overset{20}{\underset{j=1}{\sum }}P_{\mathrm{ij}}\log_{2}P_{\mathrm{ij}}$$
  where $P_{ij}$ is termed as the frequency of amino acid *j* at position *i*. The conservation score indicates the variability of residues at each position in the sequence. A value that is small at a position means that the residue is conserved.
6. Physicochemical feature. The eight physicochemical features can be obtained from the AAindex database [47]. The eight features are as follows: propensities, average accessible surface area, hydrophobicity, atom-based hydrophobic moment, polarity, polarizability, flexibility parameter for no rigid neighbors, and hydrophilicity.
7. Disordered regions. We used the DISOPRED [48] and DisEMBL [49] to predict each residue’s disordered regions in the protein sequence.
8. Solvent accessible area (ASA) calculated through the protein sequence. These features can be calculated by NetSurfP [50], SPIDER2 [51], ACC, and SSPro programs [52]
9. Blocks substitution matrix. The substitution probabilities and their relative frequencies of amino acid can be counted by BLOSUM62 [53].

### 2.4. Feature Selection

Feature selection is vital for the prediction of hot spots in protein–RNA complexes. Feature selection can help us remove irrelevant and redundant features [54,55,56]. In this paper, we calculated 156 candidate features in all. To select the optimal feature subset, we adopted a new two-step algorithm named McTWO to perform feature selection [57]. First we utilized minimum redundancy maximum relevance (mRMR) [58] to sort the importance of the features. The redundancy and relevance of mRMR was evaluated by mutual information (MI), which is written as follows:

(16) $$I\left(m,n\right)=\iint p\left(m,n\right)log\frac{p\left(m,n\right)}{p\left(m\right)p\left(n\right)}dmdn$$

where *m* and *n* represent two random variables, and p(m), p(n), and p(m,n) are the probabilistic density functions. By adopting the mRMR algorithm, we obtained 50 optimal features.

Second, we used the XGBoost algorithm to further select features from the top 50 via 10-fold cross-validation. We chose the first three features at random from the 50 optimal features as the original candidate features. We then adopted the method of sequential forward selection (SFS) to add the remaining ones to the three candidate features one by one based on the *R_c_* score. The *R_c_* score is termed as follows:

(17) $$R_{c}=\frac{1}{n}\overset{n}{\underset{i=1}{\sum }}(ACC_{i}+SENS_{i}+SPEC_{i}+AUC_{i})$$

where *n* represents the repeat times of 10-fold cross-validation.

**Figure 2:**
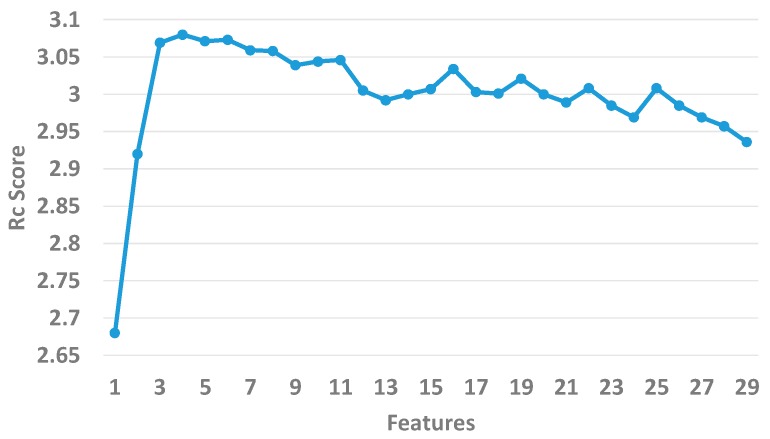
The *R_c_* values of the top 26 features.

As shown in Figure 2, we sequentially added each feature to the initial feature set and calculated the *R_c_* scores until the 26 features were put into the sets. The *R_c_* score arrives at 3.08 when the number of features is 6. The overall trend of the *R_c_* declines when the number of features continues to increase. In the end, we consider the top 6 features as optimal.

In order to evaluate the effect of the two-step feature selection algorithm, we compared it with four other extensively adopted feature selection approaches, including Boruta [59], recursive feature elimination (RFE) [60], random forest (RF) [61], and mRMR on the training dataset with 10-fold cross validation. The results are displayed in Table 2. The two-step algorithm achieved the highest value of each metric. It is obvious that the performance of the two-step algorithm is better than that of the other four methods.

### 2.5. Extreme Gradient Boosting Algorithm

The gradient boosting algorithm [62] inherits the advantages of decision trees, and it constructs an ensemble of powerful learners from weak learners. Therefore the extreme gradient boosting algorithm based on the gradient boosting algorithm makes a series of improvements concerning parallelism and predictive accuracy.

In this research, our problem was identifying hot spots and non-hot spots in protein–RNA complexes. This problem can be defined as a binary classification. We used feature vectors $F_{i}$ ($F_{i}$ = {$f_{1}$, $f_{2}$, ⋯, $f_{n}$}, i = 1, 2, ⋯, N) as input and used the class label $y_{i}$ ($y_{i}$ = {−1, +1}, i = 1, 2, ⋯, N) as the output, where N is the number of rows of the feature vectors, ’+1′ indicates hot spots, and ’-1′ represents non-hot spots. The XGBoost algorithm is a combination of classification and regression tree (CART) and a series of the gradient boosting machine [63].

### 2.6. The XGBPRH Approach

The flowchart of XGBPRH is shown in Figure 1 above. The dataset including 47 protein–RNA complexes was derived from the work of Pan et al as shown in Table 1 above. One hundred fifty-six features were generated from four sources of information: network, exposure, structure, and sequence. Next, we adopted a novel McTWO feature selection algorithm to choose the optimal features. As a result, we obtained a combination of 6 optimal features. Finally, we utilized aXGBoost classifier to predict hot spots and non-hot spots in protein–RNA complexes. 

## 3. Results

### 3.1. Assessment of Feature Importance

**Figure 3:**
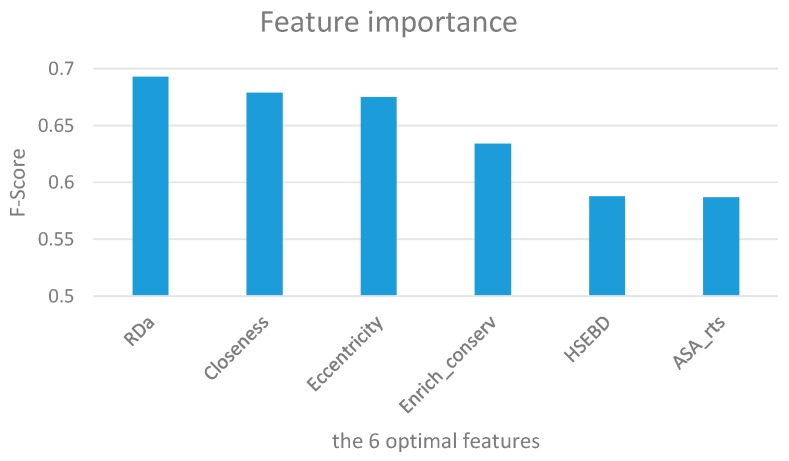
Ranking of feature importance for the six optimal features in terms of F-score.

To evaluate the relative importance of the six optimal features, we calculated the average F-score of each feature on the training dataset using XGBoost with 10-fold cross-validation. The results are summarized in Figure 3 and Table 3 over 50 trials. It is obvious that the RDa (C_a_ atom depth) feature achieves the highest F-score of 0.693. Closeness and eccentricity follow, with values of 0.679 and 0.675, respectively. This indicates that solvent exposure features and network features are vital for discriminating hot spots and non-hot spots. In our six optimal features, there are two network features (closeness and eccentricity), two exposure features (RDa and HSEBD), and two structure features (Enrich_conserv and ASA_rts).

### 3.2. Comparison of Different Machine Learing Methods

XGBPRH employs XGBoost as the classifier to determine the hot spots in protein–RNA interfaces with the six optimal features. In order to demonstrate the effectiveness of XGBoost, we used support vector machines (SVMs) [64], random forest (RF), and gradient tree boosting (GTB) to build different models and compared them with XGBPRH. Comparisons were performed with 10-fold cross validation over 50 trials according to the six optimal features. As shown in Table 4, in terms of almost all metrics, XGBoost has the best performance on the training dataset (ACC = 0.744, SENS = 0.740, SPEC = 0.755, precision = 0.785, F1-score = 0.744, MCC = 0.494, AUC = 0.822) except that the score of specificity is lower than that of the RF.

### 3.3. Performance Evaluation

**Figure 4:**
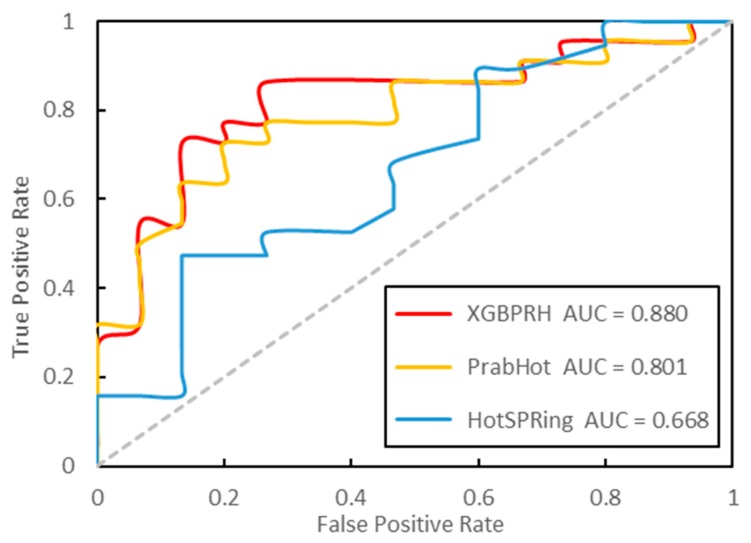
The ROC curves (receiver operating characteristic curve) of the three approaches on the independent test dataset.

As of now, there are two other hot spots prediction methods: PrabHot and HotSPRing. In order to evaluate the performance of our XGBPRH, we compared it with these. We calculated the best results and 50 repetitions’ average performance (XGBPRH-50) on the independent test dataset, respectively. As shown in Table 5 and Figure 4, the predictive performance (F1 = 0.870, MCC = 0.661, and AUC = 0.868) significantly outperforms HotSPRing and PrabHot. Moreover, the average performance over 50 trials is superior to that of PrabHot. The results prove that that our method has the best performance in predicting protein–RNA hot spot residues.

In XGBPRH, the computing time depends on the number of residues of the protein in the protein–RNA complex. Large proteins usually require more computation time than that of smaller proteins. We compared the computing time of XGBPRH with that of the PrabHot web server. The results indicate that most predictions can be finished in 5–30 min using XGBPRH. For example, a protein of 490 residues (PDB ID: 1FEU, chain A) required a calculation time of about 25 min, almost the same as PrabHot’s calculation time.

### 3.4. Case Study

#### 3.4.1. Structure of the Star Domain of Quaking Protein in Complex with RNA

**Figure 5:**
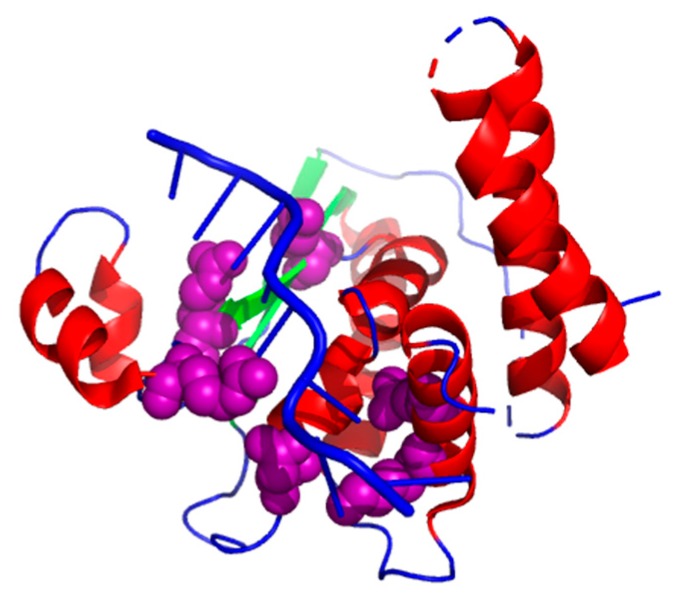
The prediction results on 4JVH using XGBPRH method. True positives colored in purple.

The complex (PDB ID: 4JVH, chain A) [65] has six hot spots (K120_A, K190_A, N97_A, Q193_A, R130_A, and R124_A). As shown in Figure 5, we chose a planned combination of colors to show the results: a helix is labeled in red, a sheet labeled in green, and a loop colored in blue. We used purple to label the true positives. It is obvious to see that our XGBPRH method correctly identified all hot spots (K120_A, K190_A, N97_A, Q193_A, R130_A, and R124_A).

#### 3.4.2. The TL5 and *Escherichia coli* 5S RNA Complex

**Figure 6:**
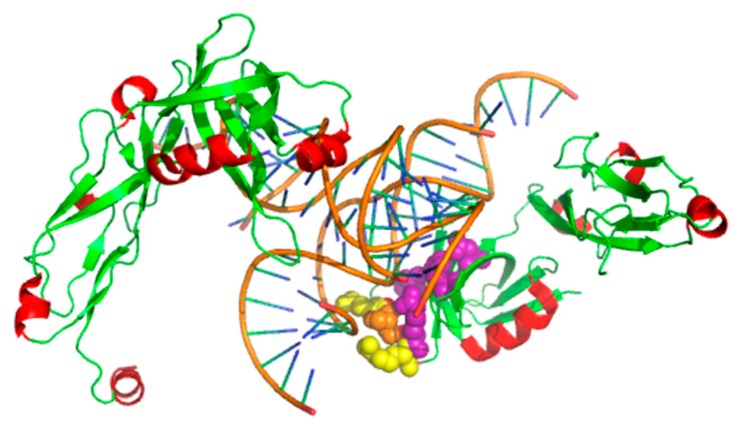
The prediction results on 1FEU using XGBPRH. True positives are labeled in purple, true negatives are labeled in yellow, and false negatives are labeled in orange.

*Thermus thermophilus* TL5 (PDB ID: 1FEU, chain A) [66] belongs to the so-called CTC family of bacterial proteins. TL5 [67] binds to the RNA with the help of its N-terminal domain. The complexes have three non-hot spots (K14_A, R20_A, and S16_A) and four hot spots (D87_E, H85_A, R10_A, and R19_A). As shown in Figure 6, our XGBPRH method correctly identified four hot spots (H85_A, R10_A, D87_E, and R19_A) and two non-hot spots (R20_A and K14_A).

## 4. Discussion

Effective prediction of protein–RNA interaction energy hotspots is of great significance in protein engineering and drug design. In this study, we combined 156 exposure, network, structural, and sequence features. To eliminate the redundant information, we utilized the McTWO feature selection algorithm combined with XGBoost to choose the most useful features, which is the difference between XGBPRH and PraHot. We demonstrated the prediction performance on the independent test dataset. The results show that XGBPRH has superior prediction accuracy. Although our method has achieved good results, there is still room for improvement. First, the protein–RNA interaction hotspot data set is still relatively small, and it is necessary to continue adding experimental data to expand the data set. Semi-supervised learning methods can also be used to improve the prediction performance using a large number of unlabeled data. Secondly, no single feature can fully identify hot spots from the protein–RNA binding interfaces. There is a need to find more effective features or feature combinations to further improve the prediction accuracy.

## Figures and Tables

**Table 1:** The dataset of 47 protein–RNA complexes (Protein DataBank [PDB] codes).

Training dataset	1ASY	1B23	1JBS	1U0B	1URN	1YVP	2BX2	2IX1
	2M8D	2PJP	2Y8W	2ZI0	2ZKO	2ZZN	3EQT	3K5Q
	3L25	3MOJ	3OL6	3VYX	4ERD	4MDX	4NGB	4NKU
	4OOG	4PMW	4QVC	4YVI	5AWH	5DNO	5IP2	5UDZ
Independent testing dataset	1FEU	1WNE	1ZDI	2KXN	2XB2	3AM1	3UZS	3VYY
	4CIO	4GOA	4JVH	4NL3	5EN1	5EV1	5HO4	

**Table 2:** The performance of the McTWO feature selection algorithm in comparison with four other feature selection algorithms.

Method	ACC	SENS	SPEC	PRE	F1	MCC	AUC
Boruta	0.65	0.603	0.733	0.733	0.634	0.337	0.730
mRMR	0.667	0.661	0.663	0.726	0.662	0.347	0.760
RFE	0.692	0.671	0.702	0.725	0.678	0.366	0.768
RF	0.708	0.698	0.727	0.767	0.711	0.435	0.821
Two-step	0.733	0.732	0.770	0.797	0.743	0.505	0.889

mRMR: Minimum redundancy maximum relevance, RFE: Recursive feature elimination, RF: Random forest, ACC: Accuracy, SENS: Sensitivity, SPEC: Specificity, PRE: Precision, F1: F1-score, MCC: Matthew’s correlation coefficient, AUC: Area under the ROC curve.

**Table 3:** The F-score of the six optimal features using XGBoost with 10-fold cross-validation over 50 trials.

Rank	Feature Name	Symbol	F-Score
1	$C_{\alpha }$ atom depth	RDa	0.693
2	Closeness	Closeness	0.679
3	Eccentricity	Eccentricity	0.675
4	Enrich conservation	Enrich_conserv	0.634
5	The number of $C_{\alpha }$ atoms in the lower half sphere	HSEBD	0.588
6	ASA (relative total_side)	ASA_rts	0.587

**Table 4:** Performance comparison of different machine learning methods.

Method	ACC	SENS	SPEC	PRE	F1	MCC	AUC
RF	0.710	0.650	0.781	0.779	0.690	0.430	0.783
SVM	0.741	0.738	0.741	0.775	0.741	0.480	0.802
GTB	0.740	0.728	0.755	0.784	0.739	0.481	0.810
XGBoost	0.744	0.740	0.755	0.785	0.744	0.494	0.822

SVM: support vector machines, GTB: Gradient Tree Boosting.

**Table 5:** Prediction performance of XGBPRH in comparison with PrabHot and HotSPRing on the independent dataset.

Method	SENS	SPEC	PRE	F1	MCC	AUC
XGBPRH	0.909	0.733	0.833	0.870	0.661	0.868
XGBPRH-50	0.880	0.537	0.739	0.802	0.454	0.817
PrabHot	0.793	0.655	0.697	0.742	0.453	0.804
PrabHot-50	0.695	0.690	0.703	0.733	0.389	0.771
HotSPRing	0.655	0.552	0.604	0.633	0.258	0.658

PrabHot: Prediction of protein–RNA binding hot spots, “-50”: 50 repetitions’ average performance of the proposed method.
